# The role of acupoint stimulation as an adjunct therapy for lung cancer: a systematic review and meta-analysis

**DOI:** 10.1186/1472-6882-13-362

**Published:** 2013-12-17

**Authors:** Hai-Yong Chen, Shi-Guang Li, William CS Cho, Zhang-Jin Zhang

**Affiliations:** 1 School of Chinese Medicine, Li Ka Shing Faculty of Medicine, The University of Hong Kong, G/F, 10 Sassoon Road, Pokfulam, Hong Kong Special Administrative Region, China; 2 Department of Oncology & Hematology, Shenzhen Hospital of TCM, Guangdong Province, China; 3 Department of Clinical Oncology, Queen Elizabeth Hospital, 30 Gascoigne Road, Kowloon, Hong Kong Special Administrative Region, China

**Keywords:** Acupuncture, Chinese medicine, Lung cancer, Systematic review, Meta-analysis

## Abstract

**Background:**

Lung cancer is the leading cause of death in cancer patients. Clinical studies showed that a variety of acupoint stimulations have been extensively used for lung cancer patients, including needle insertion, injection with herbal extraction, plaster application, and moxibustion. However, the role of acupoint stimulation in lung cancer treatment was not fully reviewed.

**Methods:**

In the present study, we conducted a systematic review and meta-analysis on the role of acupoint stimulation in lung cancer treatment by electronic and manual searching in seven databases, including Ovid (Ovid MEDLINE, AMED, CAB Abstracts, EMBASE), EBSCOhost research databases (Academic Search premier, MEDLINE, CIHAHL Plus), PreQuest (British Nursing Index, ProQuest Medical Library, ProQuest Dissertations & Theses A&I, PsycINFO), and ISI web of knowledge (Web of Science, BIOSIS Citation Index, Biological Abstracts, Chinese Science Citation Database), CNKI, Wanfang Data, and CQVIP.

**Results:**

Our study showed that acupoint stimulation has strong immunomodulatory effect for lung cancer patients as demonstrated by the significant increase of IL-2, T cell subtypes (CD3+ and CD4+, but not CD8+ cells), and natural killer cells. Further analysis revealed that acupoint stimulation remarkably alleviates the conventional therapy-induced bone marrow suppression (hemoglobin, platelet, and WBC reduction) in lung cancer patients, as well as decreases nausea and vomiting. The pooled studies also showed that acupoint stimulation can improve Karnofsky performance status, immediate tumor response, quality of life (EORCT-QLQ-C30), and pain control of cancer patients.

**Conclusions:**

Acupoint stimulation is found to be effective in lung cancer treatment, further confirmatory evaluation via large scale randomized trials is warranted.

## Background

Acupuncture has been widely used for more than three thousand years in China. It is one of the key treatment modalities in traditional Chinese medicine (TCM), which is also based on the Yin-Yang, Channel and Collateral Theories. Accordingly to TCM theories, Yin-Yang imbalance is the basis of diseases and stimulation of certain acupoints along the collaterals can nurture the qi (or vital energy) and rebalance Yin-Yang in the body. Recently, acupuncture has been widely developed into a variety form of acupoint stimulation, including needle insertion, injection with herbal extract, plaster application, and moxibustion, etc. [1]. Previous studies have shown that acupoint stimulation can be used to treat a variety of diseases and symptoms, e.g. insomnia [2], depression [3], and pain [4]. In recent decades, TCM is regarded as a complementary therapy to cancer patients worldwide [5-7]. A number of literatures have reported that acupoint stimulation may be effective on symptom management [8,9], reduction of chemotherapy-induced side effects [10-12], and quality of life improvement [13] in cancer patients.

Lung cancer is a leading cause of cancer mortality with 1.37 million deaths in 2008 worldwide. It is the most prevalent cancer in male and the fourth prevalent cancer in female. Conventional therapies for lung cancer include surgery, radiotherapy, chemotherapy, and targeted therapies (e.g. erlotinib and bevacizumab). Recently, the use of alternative and complementary therapies is increasingly widespread [14,15]. We have previously found that Chinese herbal medicine, as an adjunct therapy, has advantage in the reduction of side effects and improvement of symptoms in patients with non-small cell lung carcinoma [16]. Some clinical studies have also reported the use of acupoint stimulation as a treatment for lung cancer [17,18]. However, the role of acupoint stimulation in treating lung cancer is not thoroughly evaluated. Thus, we conducted a systematic review and meta-analysis on the efficacy of acupoint stimulation for lung cancer patients in the present study.

## Methods

### Selection criteria

Included studies have to meet all of the following criteria:

1. Studies claimed as random allocation or showed the baseline data without significant difference (age, gender, and severity) among the intervention and control groups.

2. Studies had to use acupoint stimulation as the adjunct intervention, or had to use acupoint stimulation as the primary studying objective or evaluating purpose.

3. Studies had at least one control group with conventional therapies, placebo, or other appropriate controls.

4. Studies investigated at least one of the outcomes of interest listed below:

i) Immunomodulation: changes in CD3, CD4, CD8 levels of T cell, natural killer (NK) cells, and IL-2 levels.

ii) Bone marrow suppression: changes in hemoglobin, platelets, and white blood cells (WBCs).

iii) Conventional therapy-induced side effect: nausea and vomiting. Judgment of vomiting grade was based on WHO toxicity reaction: grade 0: no nausea and vomiting; grade I: nausea; grade II: casual vomiting, not requiring medication; grade III: frequent vomiting, requiring medication; grade IV: serious vomiting, uncontrolled with medication.

iv) Immediate tumor response: number of patients with complete response (CR) or partial response (PR) evaluated with the WHO scale.

v) Performance status: the changes of Karnofsky performance status (KPS) scores.

vi) Other quality of life assessments, e.g. EORTC-QLQ-C30.

### Databases

Four major search engines were retrieved, including:

1. OVID® (Ovid MEDLINE 1946 to January 2013, AMED 1985 to 2013, CAB Abstracts 1910 to January 2013, EMBASE 1996 to February 2013).

2. EBSCOhost research databases (Academic Search premier, MEDLINE, CIHAHL Plus, all to January 2013).

3. PreQuest (British Nursing Index 1994 to January 2013, ProQuest Medical Library from starts to January 2013, ProQuest Dissertations & Theses A&I from starts to January 2013, PsycINFO 1806 to January 2013).

4. ISI web of knowledge (Web of Science® 1956 to January 2013, BIOSIS Citation Index 2006 to January 2013, Biological Abstracts® 1980 to 2012, Chinese Science Citation Database 1989 to January 2013).

The Chinese electronic databases were as the follows:

1. CNKI (China Academic Journals Full-text Database 1979 to January 2013, China Doctoral Dissertations Full-text Database 1984 to January 2013, China Masters’ Theses Full-text Database 1984 to January 2013, China Proceedings of Conference Full-text Database 1953 to January 2013).

2. Wanfang Data (1990 to January 2013).

3. CQVIP (1989 to January 2013).

### Search strategy

Electronic databases were searched using the following strategy:

Searching terms: (acupuncture OR acupressure OR acupoint OR massage OR meridian OR moxibustion OR moxa) AND (pulmonary cancer OR pulmonary carcinoma OR pulmonary adenocarcinoma OR pulmonary squamous cell carcinomas OR pulmonary neoplasms OR pulmonary nodules OR pulmonary tumor OR lung cancer OR lung carcinoma OR lung adenocarcinoma OR lung squamous cell carcinoma OR lung neoplasms OR lung nodules OR lung tumor OR non-small-cell lung carcinoma OR non-small-cell-lung carcinoma OR NSCLC OR small-cell lung carcinoma OR small-cell-lung carcinoma OR SCLC). Chinese language database was retrieved with similar search strategy. “AND” and “OR” are Boolean operators.

### Risk bias assessment

Risk bias of studies was assessed using the Cochrane Risk of Bias Assessment Tool (http://handbook.cochrane.org/, part 2, chapter 8). All trials were reviewed by at least two reviewers (HYC and SGL) and any disagreement was resolved through the involvement of a third reviewer in consensus conferences.

### Data synthesis

All analyses were performed with RevMan version 5.2 to quantify and compare the efficacy outcomes of the treatment versus the control groups. Dichotomous data were reported as relative ratio (RR) whereas continuous data were reported as standardized mean difference (SMD) ± standard deviation (SD). The random-effect model was employed when the study of heterogeneity (*I*^2^) was large than 50%, otherwise a fix-effect model was used when the *I*^2^ was less than 50%. To test the heterogeneity, subgroup analysis was performed according to the types of acupoint stimulation (needle insertion, acupuncture injection with herbs, acupoint plaster application, and moxibustion). The *Z*-test was used to compare the overall effects of the treatment groups and the control groups, differences were considered to be statistically significant when *P* < 0.05.

## Results and discussion

**Figure 1:**
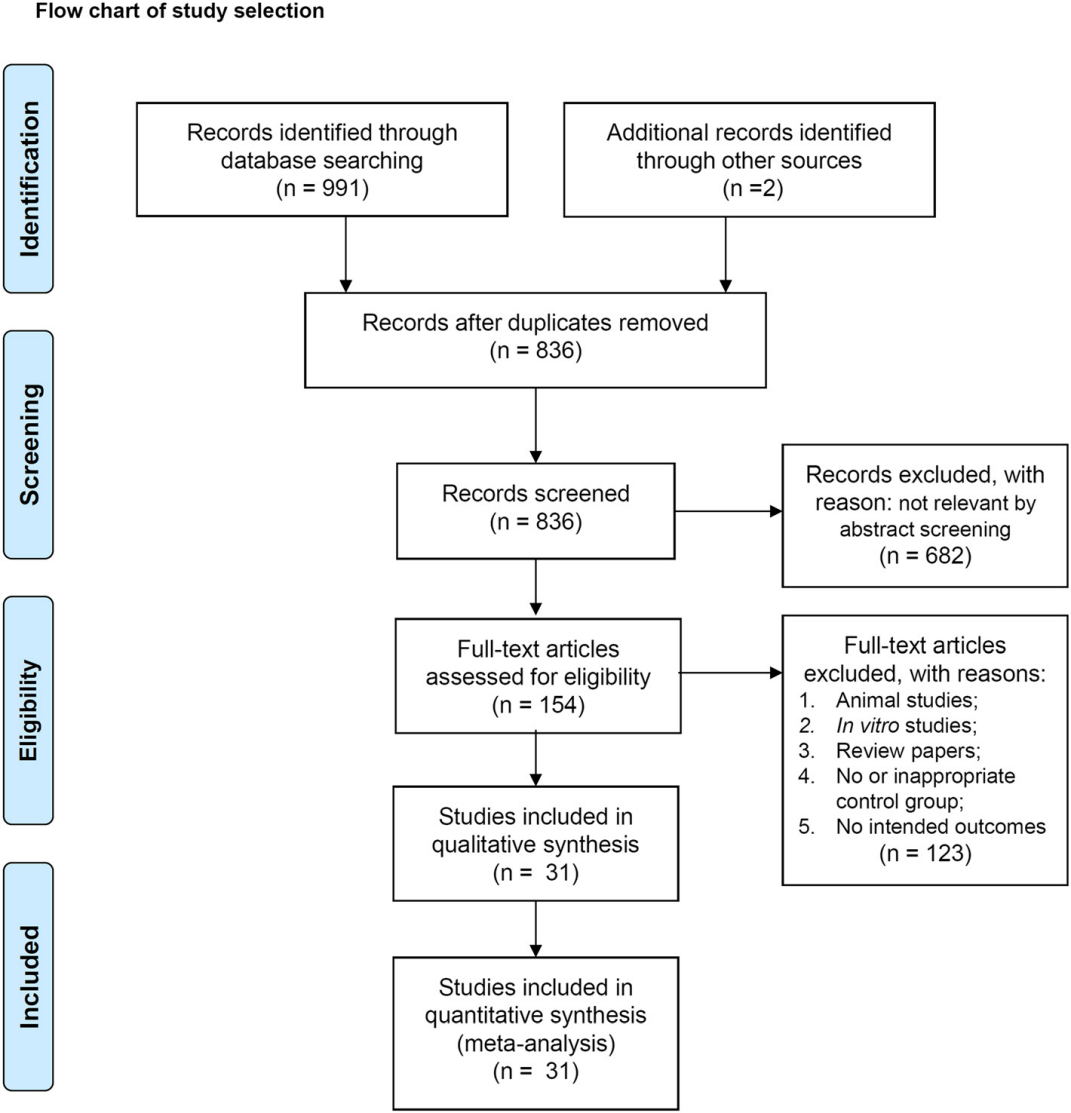
Flow chart of study selection.

**Figure 2:**
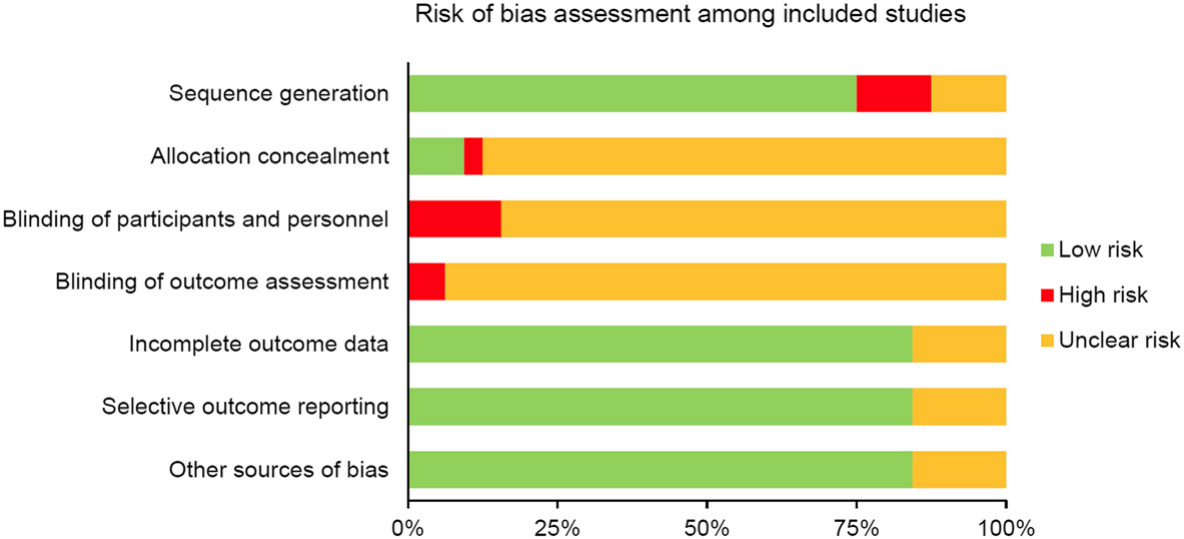
Risk of bias assessment among included studies.

993 abstracts were retrieved with 155 duplications. 154 studies in full text were further examined. A total of 31 studies satisfied the selection criteria and were analyzed in the present study (Figure 1) and their characteristics are listed in Table 1. Acupoint stimulation varies from needle insertion, pressure, plaster application, and moxibustion to herbal extraction injection on the acupoints. The most commonly used acupoints were listed in Table 2. The risk of bias in the included studies was assessed as shown in Figure 2. The risk of bias in each study was shown in (Additional file 1: Table S1).

**Table 1:** Characteristics of included studies

**Studies**	**No. of patients**	**Acupuncture group**		**Control group intervention**	**Assessment of outcomes**	**Duration**
		**Acupoints**	**Intervention**			
Cai and Wu [19]	80	Fixed points: ST36	Acupuncture + GP and Ondansetrontdn Dexamethasone	GP and Ondansetron Dexamethasone	Nausea and vomiting	8 weeks
Chen [20]	32	Fixed points: ST36 and BL23	Astragalus injection in acupuncture points + NP/CE and Huangqilifei decoction	NP/CE and Huangqilifei decoction	Tumor response; immunomodulation (CD3+, CD4+, CD4/CD8); KPS; survival rate; chemotoxicity	8 weeks
Chen [21]	60	Fixed points: BL13, LU1, LU9, ST36, PC6, etc.	Acupuncture + TP/GP and Chinese herbs by syndrome differentiation	TP/GP and Chinese herbs by syndrome differentiation	Tumor response; clinical symptom improvement; immunomodulation	8 weeks
Chen [22]	60	Fixed points: RN4	Ginger moxibustion + general nursing care	General nursing care	Sleep quality and WBCs	10 days
Chen et al. [23]	50	Fixed points: LU9, PC6, ST36, BL23, DU14	Acupuncture + CAP/EP	CAP/EP	NK and leukocyte cells	8 weeks
Ding [24]a	86	Fixed points : LU9, BL13, ST36, ST40, SP3, BL20, BL43 (bilateral)	Acupuncture + Fu zhen gan fei decoction	Fu zhen gan fei Decoction	Clinical symptom improvement; WBCs; Hb; Plt; KPS	8 weeks
Ding [25]b	32	Fixed points: ST36, PC6	Acupuncture + cis-platinum based chemotherapy and ondansetron	Cis-platinum based chemotherapy and ondansetron	Nausea and vomiting	4 weeks
Fan & Wei [26]	80	Fixed points: ST36	Vitamin B6 acupoint injection + NP/TP chemotherapy combined with ondansetron	NP/TP chemotherapy combined with ondansetron	Nausea and vomiting; KPS	Not reported
Gu et al. [27]	40	Fixed points: ST36, RN4, BL23, DU4	Acupoint plaster application + Chinese herbs (unclear ingredient)	Chinese herbs (unclear ingredient)	Immunomodulation (CD3+, CD4+, CD4/CD8), IL-2	8 weeks
He & Lou [28]	49	PC6, LI10, SP46, SP4 (bilateral); RN12 (unilateral); auricular point: liver, spleen, shen men, jiao gan	Acupoint stimulation + EP with ondansetron	EP with ondansetron	Nausea and vomiting; chemotoxicity	3 days
Huang [29]	40	LU9, BL13, BL17, ST36 (bilateral)	Crude herb moxibustion (Semen Brassicae, Manchurian Wildginger, Ephedra sinica Stapf ) + cis-platinum based chemotherapy	Cis-platinum based chemotherapy	Chemotoxicity; living quality; body weight; clinical symptoms; tumor size; immunomodulation (CD3+, CD4+, CD4/CD8)	6 weeks
Huang et al. [30]	80	PC6, ST21, ST36 (bilateral)	Acupoint plaster application + NP/GP with ondansetron	NP/GP with ondansetron	Survival; KPS; clinical symptom improvement; chemotoxicity	3 weeks
Jiang et al. [31]	43	BL23, ST36 (bilateral); DU4, RN4 (unilateral)	Acupoint plaster application decoction (of herbal medicine) + gemcitabine/ pemetrexed/docetaxel	Gemcitabine/pemetrexed/docetaxel	Time to progression (TTP); quality of life	8 weeks
Li [32]	60	BL13, BL15, BL17 (bilateral)	Acupoint plaster application with decoction (of Semen Brassicae, Manchurian Wildginger, Ephedra sinica Stapf) + Diprophylline	Diprophylline	Dyspnea valuated by numeric scale	7 days
Lin et al. [33]	83	BL17 (bilateral)	Acupuncture + NP/EP	NP/EP	Chemotoxicity (reduction of WBCs, Hb, Plt) ; KPS	10 days
Lin et al. [34]	80	four flowers acupoints: BL17, BL19 (bilateral)	Moxibustion + NP with Granisetron hydrochloride	NP with Granisetron Hydrochloride	Chemotoxicity (reduction of WBCs, Hb, Plt)	10 days
Lin [34]	60	ST36, BL13 (bilateral)	Acupoint injection (Chuan ke zhi) + Aminophylline injection	Aminophylline injection	KPS; immunomodulation (CD3+, CD4+); clinical symptoms improvement	2 weeks
Liu & Wang [35]	60	RN12	Acupoint plaster application with herbal medicine (Pinellia Tuber and Syzygium aromaticum, etc.) + cis-platinum based chemotherapy + Granisetron hydrochloride injection	Cis-platinum based chemotherapy + Granisetron Hydrochloride injection	KPS; appetite score	3 days
Lou [36]	51	bilateral: ST36, RN4, LU5	Acupoint magnet + GP	GP	Immunomodulation (CD3+, CD4+, CD4/CD8)	2 weeks
Ou Yang et al. [37]	69	RN8	Moxibustion with salt + NP/GP	NP/GP	Clinical symptom improvement; immunomodulation (CD3+, CD4+, CD11+); nausea and vomiting	4 ~ 8 weeks
Qiao et al. [38]	56	Ashi acupoints, ST36, KI1	Millimeter wave treatment + Gu se fang granules	Gu se fang granules	Clinical symptom improvement; KPS	4 weeks
Shi et al. [39]	32	ST36, BL23, BL20, RN6, RN4, LU10, LI10, LI4, SI3, PC4, PC6, SJ6 (bilateral)	Acupuncture + general anesthesia	General anesthesia	Immunomodulation (CD3+, CD4+, CD4/CD8)	Not reported
Tao et al. [40]	100	ST36, PC6 (bilateral); RN4	Acupoint plaster application + chemotherapy with metoclopramide or ondansetron	Chemotherapy with metoclopramide or ondansetron	Nausea and vomiting	7 days
Wang [41]	60	Four flowers acupoints: BL17, BL19 (bilateral)	Moxibustion + NP	NP	TNF-α, IL-2, WBCs; nausea and vomiting; KPS	7 days
Xu [42]	60	Four flowers acupoints: BL17, BL19 (bilateral)	Fire needle therapy + GP/DP combined with ondansetron	GP/DP combined with ondansetron	Immunomodulation (CD3+, CD4+, CD4/CD8); TNF-α, IL-2; KPS	7 days
Xu et al. [43]	45	ST36, DU14	Acupoint injection (Radix Sophorae Flavescentis extraction) + anti-tumor Chinese herbs	Anti-tumor Chinese herbs	KPS; blood cells; chemotoxicity (WBCs, Hb, Plt)	4 weeks
Xuan et al. [44]	60	BL13 (bilateral); DU14	Acupoint plaster application + traditional Chinese medicine	Traditional Chinese medicine	Immunomodulation (CD3+, CD4+, CD4/CD8); KPS	4 weeks
Zhang [45]	60	Four flowers acupoints: BL17, BL19 (bilateral)	Moxibustion + GP/DP combined with ondansetron	GP/DP combined with ondansetron	Immunomodulation (CD3+, CD4+, CD4/CD8), TNF-α, IL-2, CSF; KPS	7 days
Zhang & Cheng. [46]	40	ST36, BL13	Chuan ke zhi injection combined with Aminophylline injection to acupoint + routine treatment for symptoms	Routine treatment for symptoms	Lung function, clinical symptoms; immunomodulation (CD3+, CD4+, CD4/CD8)	2 weeks
Zhou et al. [47]	15	ST36, BL23, BL20, RN6, RN4, LU10, LI10, LI4, SI3, PC4, PC6, SJ6 (bilateral)	Acupuncture + general anesthesia	General anesthesia	Immunomodulation (CD3+, CD4+, CD4/CD8)	Not reported
Zhou et al. [48]	35	Acupoint selection treatment based on syndrome differentiation	Abdominal acupuncture + routine treatment for symptoms	Routine treatment for symptoms	KPS, EORTC-QLQ-C30	4 weeks

CAP, cyclophosphamide + adriamycin + cisplatinum; CE, carboplatin and etoposide; CSF, colony stimulating factors; DP, docetaxel + cisplatinum; EP, VP-16 + cisplatinum; GP, gemcitabine + cisplatinum; Hb, hemoglobin; KPS, Karnofsky performance score; NP, vinorelbine + cisplatinum; Plt, platelet; TP, paclitaxel + cisplatinum; WBCs, white blood cells.

**Table 2:** Commonly used acupoints

**Acupoints**	**Counts**	**Frequency (%)**
ST36 (Zu San Li )	19	64.5
PC6 (Nei Guan)	8	25.8
BL17 (Ge Shu)	8	25.8
BL13 (Fei Shu)	7	22.6
RN4 (Guan Yuan)	7	22.6
BL23 (Shen Shu)	6	19.4

### Immunomodulation

**Figure 3:**
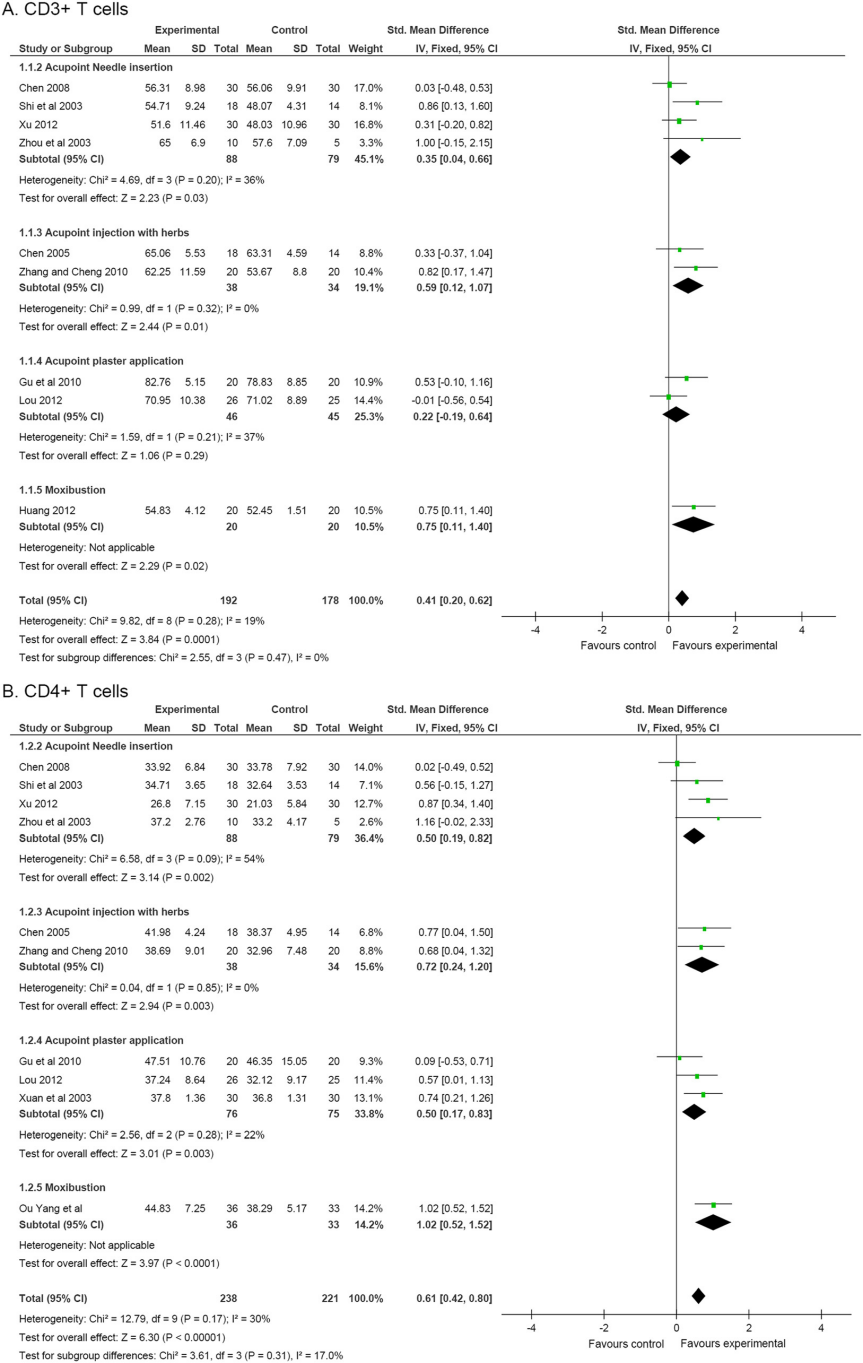
**Immunomodulation of acupuncture in lung cancer patients. (A)** CD3+ T cells; **(B)** CD4+ T cells.

A remarkable increase in CD3+ T cell level was reported in patients treated with acupoint stimulation (SMD, 0.41 [95% CI, 0.20 to 0.62], *P* = 1E-4, 9 studies, 370 patients) (Figure 3A), and the heterogeneity test indicated no significant difference among those studies [20,21,27,29,36,39,42,46],[47]. Subgroup analysis showed that acupoint needle insertion (SMD, 0.35 [95% CI, 0.04 to 0.66], *P* = 0.03, 4 studies) and acupoint injection with herbs (SMD, 0.59 [95% CI, 0.12 to 1.07], *P* = 0.01, 2 studies) had advantage in improving CD3+ while acupoint plaster application (SMD, 0.22 [95% CI, -0.19 to 0.64], *P* = 0.29, 2 studies) had no significant advantage in CD3+ improvement.

We also observed an improvement in the CD4+ T cell level (SMD, 0.61 [95% CI, 0.42 to 0.80], *P* < 1E-5, 10 studies, 459 patients) (Figure 3B) [20,21,27,36,37,39,42,44],[46,47]. Subgroup analysis showed that acupoint needle insertion (SMD, 0.50 [95% CI, 0.19 to 0.82], *P* = 0.002, 4 studies) and acupoint injection with herbs (SMD, 0.72 [95% CI, 0.17 to 0.83], *P* = 0.003, 2 studies) had advantage in improving CD4+ while acupoint plaster application (SMD, 0.50 [95% CI, 0.17 to 0.83], *P* = 0.003, 3 studies) had no significant advantage in CD4+ improvement.

The CD8+ T cell level in patients treated with acupuncture has shown no significant difference compared with the control group (SMD, 0.0 [95% CI, -0.19 to 0.19], *P* = 1.0, 10 studies, 459 patients) (Additional file 2: Figure S1A) [20,21,27,36,37,39,42,44],[46,47]. Subgroup analysis showed that acupoint injection with herbs (SMD = −0.67, 95% CI = -1.15 to -0.20, p = 0.006, 2 studies) had advantage in lowing CD8+, acupoint plaster application (SMD, 0.21 [95% CI, -0.18 to 0.61], *P* = 0.29, 2 studies) had advantage in upregulating CD8+, and acupoint needle insertion (SMD, 0.0 [95% CI, -0.31 to 0.31], *P* = 0.99, 4 studies) had no significant advantage. Heterogeneity test indicated a significant difference among the acupoint needle insertion subgroup. After further removing any study among them, the acupoint needle insertion group still showed no significant alternation compared with the control groups.

As shown in Additional file 2: Figure S1B, the pooled studies indicated that acupoint stimulation can increase chemotherapy-induced NK cell reduction compared to the control group (SMD, 0.59 [95% CI, 0.21 to 0.97], *P* = 0.002, 3 studies, 114 patients) [20,23,47]. Among them, two studies used needle insertion and the left used acupoint injection with herb extraction.

Four studies used IL-2 as the outcome measurement to assess the efficacy of acupoint stimulation as an adjunct therapy for lung cancer [27,41,42,45]. The acupoint stimulation group showed a slightly better outcome than the control (SMD, 0.28 [95% CI, 0.01 to 0.55], *P* = 0.04, 4 studies, 220 patients) (Additional file 2: Figure S1C).

There was no significant difference in the baseline of CD3+, CD4+, CD8+ T cells, NK cells, and IL-2 between the acupoint stimulation and control groups as shown in Table 3.

**Table 3:** Baseline of included studies

**Index**	**Standard mean difference 95% CI**	**Heterogeneity**	**Overall effect (*P *** **value)**
***Immunomodulation***			
CD3+ T cells	0.07 [−0.30, 0.44 ]	*I*^2^ = 67%	*Z* = 0.35 (*P* = 0.72)
CD4+ T cells	−0.04 [−0.31, 0.24]	*I*^2^ = 52%	*Z* = 0.27 (*P* = 0.79)
CD8+ T cells	−0.01 [−0.19, 0.18]	*I*^2^ = 0%	*Z* = 0.08 (*P* = 0.94)
Natural killer cells	−0.38 [−1.39, 0.62]	*I*^2^ = 79%	*Z* = 0.75 (*P* = 0.45)
IL-2	0.05 [−0.21, 0.32]	*I*^2^ = 0%	*Z* = 0.39 (*P* = 0.70)
***Bone marrow suppression***			
Hemoglobin	0.04 [−0.19, 0.27]	*I*^2^ = 0%	*Z* = 0.37 (*P* = 0.71)
Platelets	0.06 [−0.17, 0.29]	*I*^2^ = 0%	*Z* = 0.53 (*P* = 0.60)
White blood cells	0.07 [−0.11, 0.24 ]	*I*^2^ = 0%	*Z* = 0.75 (*P* = 0.45)
***Clinical efficacy***			
Karnofsky performance score	−0.08 [−0.25, 0.09]	*I*^2^ = 0%	*Z* = 0.93 (*P* = 0.35)

### Bone marrow suppression

The pooled study showed that the prevention against hemoglobin reduction was significantly in favor of the acupoint stimulation group (SMD, 0.40 [95% CI, 0.17 to 0.63], *P* = 7E-4, 5 studies, 296 patients) (Additional file 2: Figure S2A) [24,42,43,45,49]. There was no significant heterogeneity among these studies (*P* = 0. 64).

The number of patients with decreased platelets was significantly reduced in the acupoint stimulation group (SMD, 0.28 [95% CI, 0.05 to 0.51], *P* = 0.02, 5 studies, 296 patients) (Additional file 2: Figure S2B) [24,42,43,45,49].

The inhibition of WBCs in lung cancer patients with acupoint stimulation was significant reduced (SMD, 0.93 [95% CI, 0.44 to 1.42], *P* < 2E-4, 8 studies, 519 patients) [22,24,33,34,42,43,45,49], but there was a prominent heterogeneity among these studies (*P* = 1E-5) (Additional file 2: Figure S2C). Sensitivity test showed that removing any of the studies did not alter the patterns, however, there is still heterogeneity among the studies (data not shown).

As shown in Table 3, there was no significant difference in the baseline of hemoglobin, platelets, and WBCs between the acupoint stimulation and control groups.

### Nausea and vomiting

**Figure 4:**
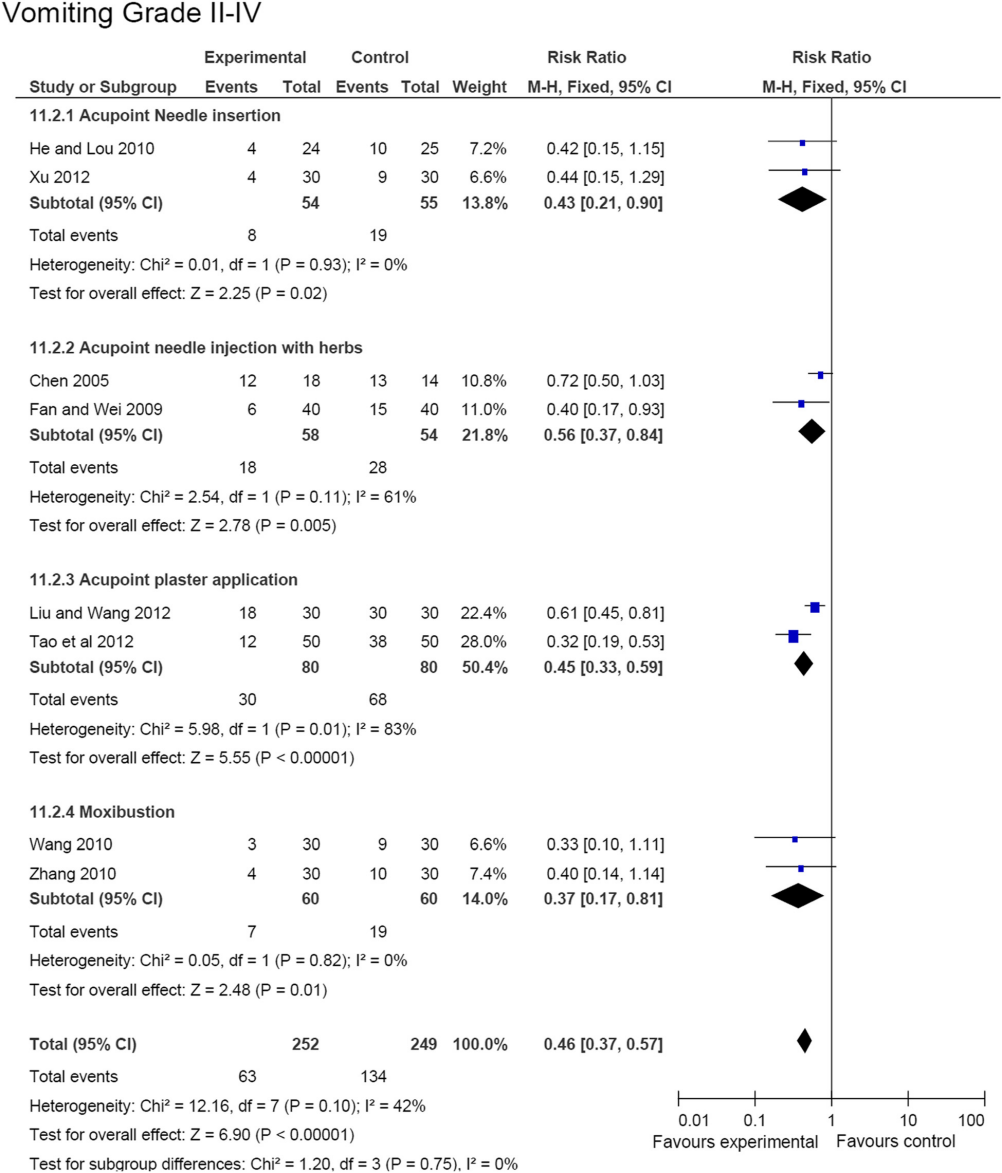
Effective responses of nausea and vomiting (vomiting grade II to IV).

As shown in Figure 4, the occurrence of chemotherapy-induced nausea and vomiting at Grade II-IV was remarkably reduced in the acupoint stimulation group compared to the control group (RR, 0.46 [95% CI, 0.37-0.51], *P* = 1E-5, 8 studies, 501 patients) [19,20,25,26,28,41,42,45]. Subgroup analysis showed that acupoint needle insertion, acupoint injection with herbs, and moxibustion significantly attenuated the grade of nausea and vomiting (*P* = 0.02, *P* = 0.005, and *P* = 0.01, respectively).

### Clinical efficacy

**Figure 5:**
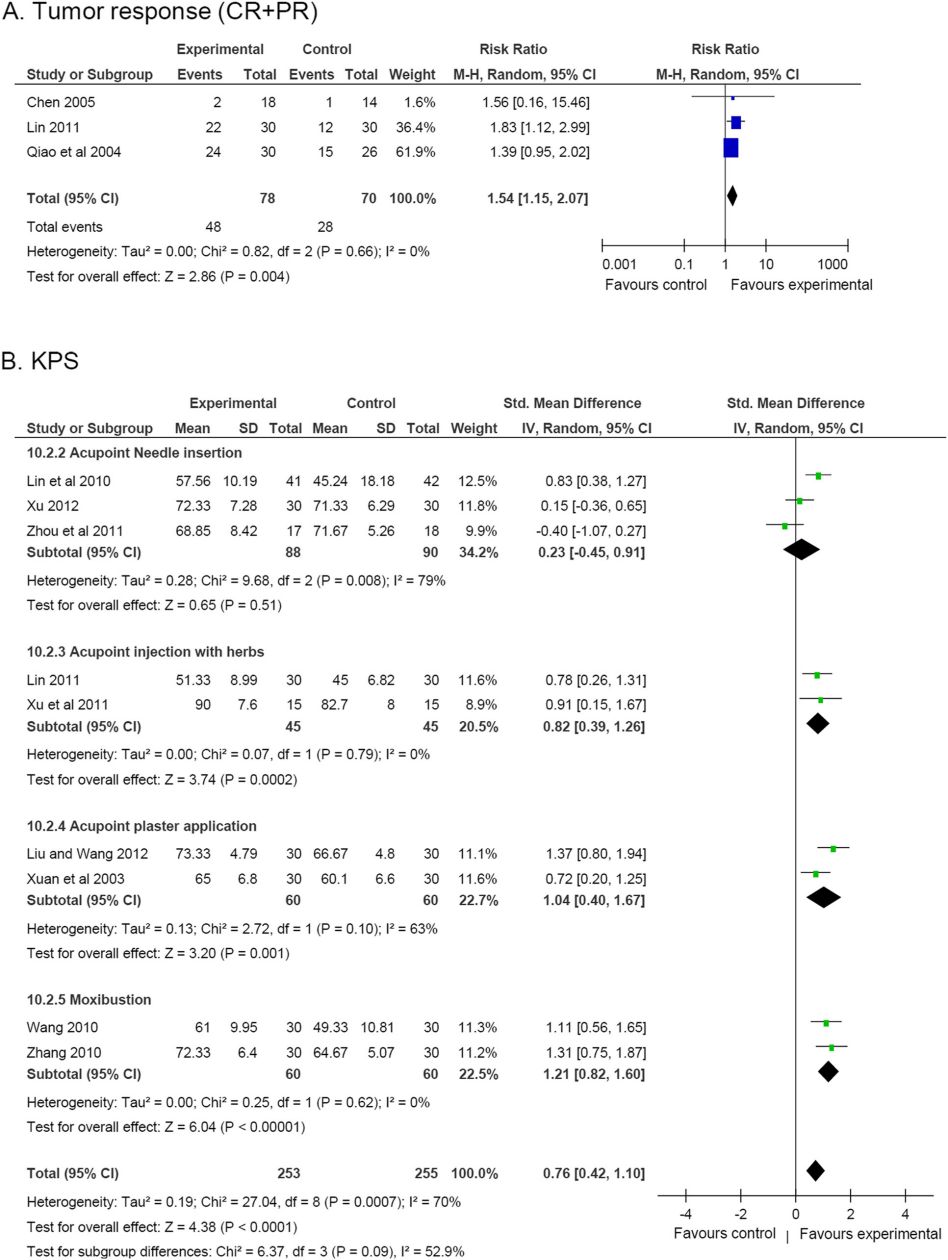
**Clinical efficacy. (A)** Tumor response; **(B)** KPS.

The immediate tumor response indicated that acupoint stimulation had a significant advantage compared to the control group (RR, 1.54 [95% CI, 1.15 to 2.07], *P* = 0.004, 3 studies, 148 patients) (Figure 5A) [20,38,49]. Two studies used acupoint injection with herb extraction and one used microwave treatment in the assessed studies.

The pooled KPS Scale showed a significant increase of clinical performance in the acupoint stimulation group compared to the control group (SMD, 0.76 [95% CI, 0.42 to 1.10], *P* < 1E-4, 9 studies, 508 patients) as shown in Figure 5B [25,33,38,41,42,44,45,48],[49]. However, the heterogeneity study showed a significant difference among these studies. Sensitivity test indicated a significant increase of KPS in the acupoint stimulation group with removal of anyone study in the nine studies. Subgroup analysis showed that acupoint injection with herb extraction, plaster application, and moxibustion had significant advantage in KPS without heterogeneity in studies (*P* = 0.0002, *P* = 0.001, and *P* < 0.0001 respectively). Acupoint needle insertion also had no advantage in KPS, but Zhou et al.’s study in the needle insertion group showed a high heterogeneity (*P* = 0.51, *I*^2^ = 79%) compared to the other two studies [48]. The baseline of KPS showed no significant difference between the acupoint stimulation group and the control group (Table 3).

**Figure 6:**
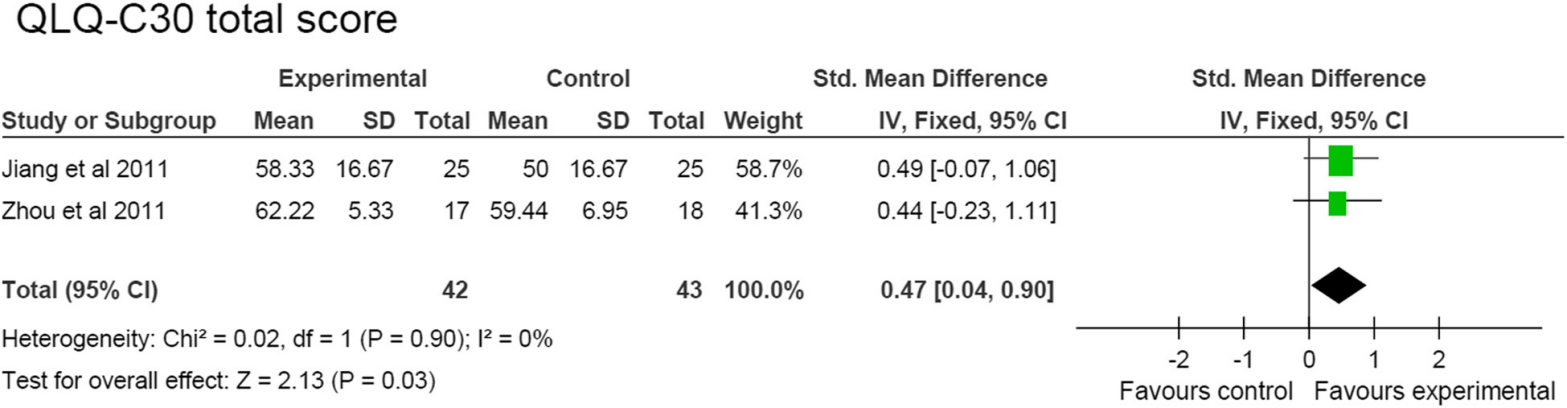
**Quality of life assessments.** QLQ-C30 total score.

EORTC-QLQ-C30 also showed a total favorable score in the acupoint stimulation group compared to the control group (SMD, 0.47 [95% CI, 0.04 to 0.90], *P* = 0.03, 2 studies, 85 patients) as shown in Figure 6A [31,48]. In addition, the Visual Analog Scale had a significant improvement in the acupoint stimulation group compared to the control group (SMD, -1.13 [95% CI, -1.58 to −0.69], *P* < 1E-5, 2 studies, 92 patients) [20,32] (Figure 6).

## Discussion

In the present study, we systematically reviewed the role of acupoint stimulation in lung cancer management. Our results showed that acupoint stimulation has immunomodulatory effect for lung cancer patients, which was demonstrated by a significant increase of IL-2, CD3+ and CD4+ T cells, NK cells, but not CD8+ T cells. Further analysis also revealed that acupoint stimulation remarkably reduces the conventional therapy-induced bone marrow suppression, enhances hemoglobin and platelets in lung cancer patients, and decreases the chemotherapy-induced nausea and vomiting. In addition, the pooled studies also showed that acupoint stimulation has an advantage in the improvement of performance status, immediate tumor response, and quality of life (EORCT-QLQ-C30).

We found that acupoint stimulation enhances T cell subtype CD3+ and CD4+ cells, but not CD8+ cells. Subgroup analysis indicated that acupoint insertion and injection with herb extraction are able to elevate the total T cells (CD3+) and T helper cells (CD4+) in lung cancer patients. Acupoint plaster application enhances CD4+ T cells, but not CD3+ T cells. CD8+ is cytotoxic T cells which are one of the most effective immune cells to kill tumor cells [50]. Interestingly, our study showed that acupoint stimulation has no significant effect on the increase of CD8+ T cells compared to the control group. Subgroup analysis showed that acupoint needle insertion has no significant effect, while acupoint injection with herbs decreases CD8+ T cells and plaster application increases CD8+ T cells in lung cancer patients. Although some studies have shown that acupuncture may upregulate CD8+ expression in patients [51,52], there may be various mechanisms for the immunomodulatory effects of acupoint stimulation lung cancer patients. T helper cells (CD4+) have four subtypes, including Th1, Th2, Treg, and Th-17 cells. IL-23 is a newly identified cytokine that has close association with Th17. On the other hand, IL-23 has been shown to impair antitumor CD8+ T cells and dendritic cells transduced with IL-23 have the ability to trigger strong antitumor activity [50].

More importantly, our findings have indicated that acupoint stimulation upregulates IL-2 in lung cancer patients. It is in line with another study that acupuncture can enhance IL-2 expression by stimulation of acupoint ST36 in rats [53]. As shown in Table 2, ST36 is the most common acupoint used for lung cancer patients. Previous study has shown that IL-2 is necessary for the growth, proliferation, and differentiation of T cells [54]. A recent study has also demonstrated that IL-2 controls the balance between Th-17 and Treg cells in the tumor microenvironment [55]. It has been hypothesized that acupuncture may also upregulate NK cells in lung cancer patients. A number of studies have demonstrated that acupuncture is a strong immunomodulator of NK cells in animals and human [56-60]. Upregulation of NK cells may contribute to the antitumor effect in cancer patients [61].

Anemia, thrombocytopenia, and pancytopenia are the most common symptoms of bone marrow suppression during chemotherapy or radiotherapy. Our findings indicated that acupuncture reduces conventional therapy-induced bone marrow suppression, e.g. increase of WBCs, hemoglobin, and platelets, which is in accordance with other findings that acupuncture can improve bone marrow suppression during chemotherapy [62]. Although the mechanism remains largely unknown, previous studies have provided some possible mechanisms, for examples, acupuncture induces the erythropoietin expression [63] and bone marrow megakaryocytes [62]. Moreover, upregulation of IL-2 by acupuncture promotes the mature of T cells [54]. Subgroup analysis showed that acupoint needle insertion, injection with herb extraction, and moxibustion may play different roles in modulating WBCs, hemoglobin, and platelets.

Reduction of side effects (SEs) is the main goal for using commentary therapies during conventional cancer treatments. Our previous studies have shown that Chinese herbal medicine has the advantage of minimizing chemotherapy-induced SEs in colon cancer [7], nasopharyngeal carcinoma [6], hepatocellular carcinoma [5], and non-small cell lung cancer [16]. These studies showed an alleviation of nausea and vomiting during TCM treatment, which is in line with our present findings that acupoint stimulation may reduce nausea and vomiting during lung cancer treatment. As acupoint stimulation has a neuromodulatory effect on GI motility and the mechanism involving endogenous opiates, it may be effective in treating chemotherapy-induced nausea and vomiting [64,65]. On the other hand, pain, cough, constipation, hair and weight loss are also common SEs during lung cancer treatment. However, no pooled data was analyzed due to the lack of relevant clinical studies. Therefore, whether acupoint stimulation can reduce these SEs during lung cancer treatment needs to be further explored.

Our finding also showed the enhancement of immediate tumor response, suggesting that acupoint stimulation may improve clinical symptoms and performance status. KPS analysis showed that acupoint stimulation (acupoint injection, plaster application, and moxibustion) can enhance performance status during lung cancer treatment. EORTC-QLQ-C30 is a questionnaire developed to assess the quality of life of cancer patients. The analysis of EORTC-QLQ-C30 showed that acupoint stimulation has an advantage in the improvement of quality of life. However, the supplemented questionnaire QLQ-C13 for lung cancer was not analyzed in this meta-analysis as there was only one study reporting the results of QLQ-C13.

Although the current evidence indicates that acupoint stimulation plays a positive role in lung cancer treatment. However, the different forms of acupoint stimulations may play different role in immunomodulation, bone marrow suppression, reduction of SEs, and improvement of performance status as demonstrated in our studies. Nevertheless, it is no doubt that using acupoint stimulation as an adjunct therapy not only can reduce the SEs of chemotherapy and radiotherapy, but also can enhance immunomodulation, attenuate bone marrow suppression, as well as improve clinical efficacy and quality of life.

Even with these promising results, there are some limitations for the present study. The frequency and duration of treatment varied in the included studies from several days to weeks, which may lead to heterogeneity in the analyzed studies. Moreover, we did not analyze the herbs used in acupoint injection and plaster application as the main purpose of this study is to evaluate the effect of acupoint stimulation as an adjunct therapy for lung cancer. But we can’t exclude the herb effects in acupoint stimulation (acupoint injection and plaster application). In addition, there were a variety of control interventions that may also increase heterogeneity of the included studies.

Besides, the efficacy of acupoint stimulation on the prolongation of survival rate for lung cancer patients remains unexplored. Risk bias study showed that a number of studies are unclear with high risks in allocation concealment, blinding of participants and personnel, as well as outcome assessments. The unclear and high risk of bias in the included studies weakens the conclusion and well-designed randomized clinical trials are warranted to confirm the efficacy of acupoint stimulation in lung cancer. In the present study, we also conducted the sensitivity test, excluding studies with less than three of seven items marked as low risk from the risk bias assessment table. The sensitivity test (data not shown) is in line with the results from the analysis of all the included studies. Although the included literatures indicate that acupuncture is effective for symptom management, reducing SEs, and improving immune response in cancer patients, the underlining mechanism on the efficacy of acupuncture is mostly unknown and this deserves further exploration by mechanistic studies. This systematic review also arouses the need for better designed randomized trials of acupuncture for lung cancer patients to support the meaningful findings of the included studies.

## Conclusion

Acupoint stimulation is found to be effective in lung cancer treatment, further confirmatory evaluation via large scale randomized trials is warranted.

## Competing interests

The authors declare that they have no competing interests.

## Authors’ contributions

WCSC and ZJZ initiated and supervised the project. SGL and HYC retrieved the databases, extracted, analyzed data, and wrote the manuscript. SGL, HYC, WCSC, and ZJZ all involved in the conception, design, interpretation of data, as well as revision and final approval of the article. All authors read and approved the final manuscript.

## Pre-publication history

The pre-publication history for this paper can be accessed here:

http://www.biomedcentral.com/1472-6882/13/362/prepub

## Supplementary Material

Additional file 1: Table S1 Risk of bias graph for the included studies. Click here for file

Additional file 2: Figure S1 CD8+, NK cells, and IL-2 in acupuncture treatment and control group. (A) CD8+, (B) NK cells, and (C) IL-2. **Figure S2:** Bone marrow suppression in acupuncture treatment and control group. (A) Hemoglobin, (B) Platelet, and (C) White blood cell (WBC). **Figure S3:** Effective response of nausea and vomiting in treatment and control group. Click here for file
