# Supplementary figures and images for: The role of acupoint stimulation as an adjunct therapy for lung cancer: a systematic review and meta-analysis
Source: BMC Complement Altern Med. 2013 Dec 17;13:362. doi: 10.1186/1472-6882-13-362 (PMC4029525; doi:10.1186/1472-6882-13-362)

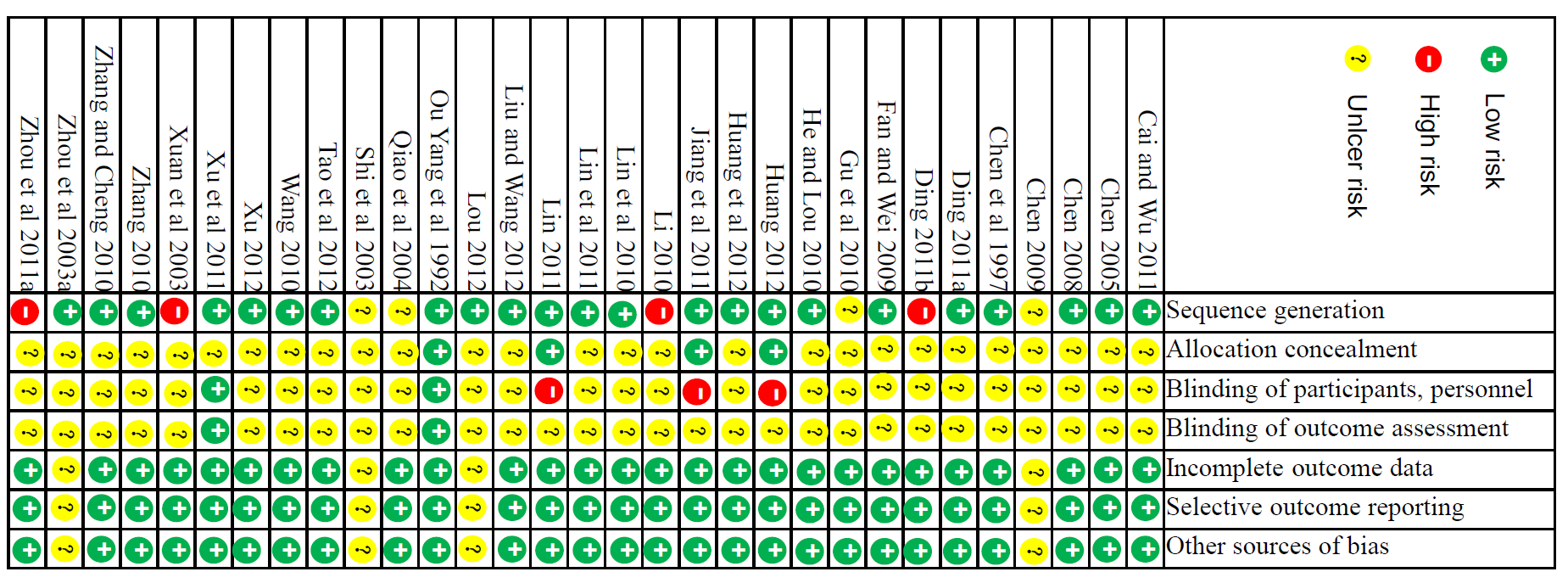

Supplement: Additional file 1: Table S1 — Risk of bias graph for the included studies. [file 1472-6882-13-362-S1.tiff]
