# Supplementary material for: The role of acupoint stimulation as an adjunct therapy for lung cancer: a systematic review and meta-analysis
Source: BMC Complement Altern Med. 2013 Dec 17;13:362. doi: 10.1186/1472-6882-13-362 (PMC4029525; doi:10.1186/1472-6882-13-362)
Supplement: Additional file 2: Figure S1 — CD8+, NK cells, and IL-2 in acupuncture treatment and control group. (A) CD8+, (B) NK cells, and (C) IL-2. Figure S2: Bone marrow suppression in acupuncture treatment and control group. (A) Hemoglobin, (B) Platelet, and (C) White blood cell (WBC). Figure S3: Effective response of nausea and vomiting in treatment and control group. [file 1472-6882-13-362-S2.pdf]

Additional file 2: supplementary figures

A. CD8+ T cells

Study or Subgroup	Experimental Mean	Experimental SD	Experimental Total	Control Mean	Control SD	Control Total	Weight	Std. Mean Difference IV, Fixed, 95% CI
**1.3.2 Acupoint Needle insertion**								
Chen 2008	25.28	4.93	30	24.61	5.12	30	13.5%	0.13 [-0.38, 0.64]
Shi et al 2003	23.09	2.9	18	22.57	3.11	14	7.1%	0.17 [-0.53, 0.87]
Xu 2012	18.1	4.56	30	20.57	5.53	30	13.1%	-0.48 [-0.99, 0.03]
Zhou et al 2003	26.2	1.83	10	22.4	3.56	5	2.3%	1.43 [0.21, 2.66]
**Subtotal (95% CI)**			**88**			**79**	**36.1%**	**-0.00 [-0.31, 0.31]**
Heterogeneity: Chi² = 9.08, df = 3 (P = 0.03); I² = 67%								
Test for overall effect: Z = 0.01 (P = 0.99)								
**1.3.3 Acupoint injection with herbs**								
Chen 2005	30.89	4.97	18	33.98	5.58	14	6.8%	-0.57 [-1.29, 0.14]
Zhang and Cheng 2010	23.54	4.42	20	27.65	6.11	20	8.4%	-0.76 [-1.40, -0.11]
**Subtotal (95% CI)**			**38**			**34**	**15.2%**	**-0.67 [-1.15, -0.20]**
Heterogeneity: Chi² = 0.14, df = 1 (P = 0.71); I² = 0%								
Test for overall effect: Z = 2.76 (P = 0.006)								
**1.3.4 Acupoint plaster application**								
Gu et al 2010	29.92	10.27	20	26	9.07	20	8.8%	0.40 [-0.23, 1.02]
Lou 2012	28.39	10.57	26	21.36	10.01	25	10.9%	0.67 [0.11, 1.24]
Xuan et al 2003	24	1.01	30	23.9	1.09	30	13.5%	0.09 [-0.41, 0.60]
**Subtotal (95% CI)**			**76**			**75**	**33.2%**	**0.36 [0.04, 0.69]**
Heterogeneity: Chi² = 2.24, df = 2 (P = 0.33); I² = 11%								
Test for overall effect: Z = 2.20 (P = 0.03)								
**1.3.5 Moxibustion**								
Ou Yang et al	37.56	6.92	36	38.34	6.42	33	15.5%	-0.12 [-0.59, 0.36]
**Subtotal (95% CI)**			**36**			**33**	**15.5%**	**-0.12 [-0.59, 0.36]**
Heterogeneity: Not applicable								
Test for overall effect: Z = 0.48 (P = 0.63)								
**Total (95% CI)**			**238**			**221**	**100.0%**	**0.00 [-0.19, 0.19]**
Heterogeneity: Chi² = 24.18, df = 9 (P = 0.004); I² = 63%								
Test for overall effect: Z = 0.00 (P = 1.00)								
Test for subgroup differences: Chi² = 12.72, df = 3 (P = 0.005), I² = 76.4%								

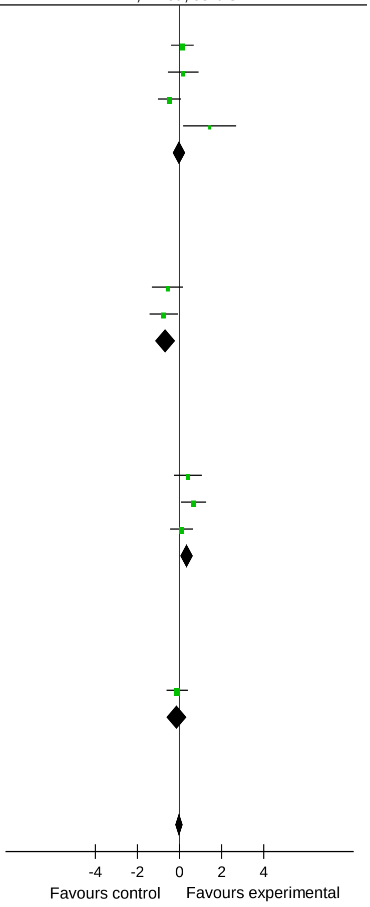

B. NK cells

Study or Subgroup	Experimental Mean	Experimental SD	Experimental Total	Control Mean	Control SD	Control Total	Weight	Std. Mean Difference IV, Fixed, 95% CI
Chen 2005	13.65	4.58	18	13.25	2.87	14	29.6%	0.10 [-0.60, 0.80]
Chen et al 1997	42.27	17.66	25	29.61	11.53	25	42.9%	0.84 [0.26, 1.42]
Zhou et al 2003	25.35	10.51	18	17.43	10.42	14	27.5%	0.74 [0.01, 1.46]
**Total (95% CI)**			**61**			**53**	**100.0%**	**0.59 [0.21, 0.97]**
Heterogeneity: Chi² = 2.74, df = 2 (P = 0.25); I² = 27%								
Test for overall effect: Z = 3.05 (P = 0.002)								

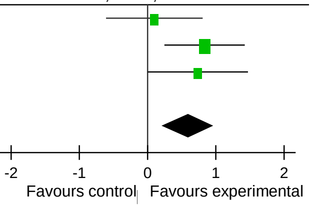

C. IL-2

Study or Subgroup	Experimental Mean	Experimental SD	Experimental Total	Control Mean	Control SD	Control Total	Weight	Std. Mean Difference IV, Fixed, 95% CI
Gu et al 2010	16.1	6.81	20	13.07	5.32	20	18.1%	0.49 [-0.14, 1.12]
Wang 2010	3.5223	1.2408	30	3.7917	1.0536	30	27.8%	-0.23 [-0.74, 0.28]
Xu 2012	4.455	0.785	30	4.197	0.906	30	27.7%	0.30 [-0.21, 0.81]
Zhang 2010	3.9237	0.9159	30	3.3577	0.7831	30	26.5%	0.66 [0.14, 1.18]
**Total (95% CI)**			**110**			**110**	**100.0%**	**0.28 [0.01, 0.55]**
Heterogeneity: Chi² = 6.31, df = 3 (P = 0.10); I² = 52%								
Test for overall effect: Z = 2.05 (P = 0.04)								

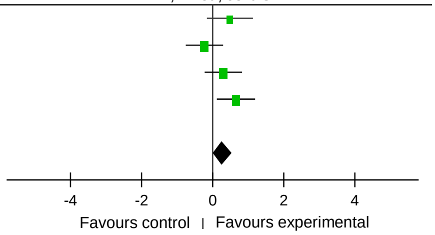

Figure S1: CD8+, NK cells, and IL-2 in acupuncture treatment and control group.

A. Hemoglobin

Study or Subgroup	Experimental Mean	Experimental SD	Experimental Total	Control Mean	Control SD	Control Total	Weight	Std. Mean Difference IV, Fixed, 95% CI
**2.1.1 Acupoint Needle insertion**								
Ding 2011a	99.09	21.95	48	94.07	20.58	38	29.3%	0.23 [-0.19, 0.66]
Xu 2012	110.27	10.39	30	108.17	9.41	30	20.8%	0.21 [-0.30, 0.72]
**Subtotal (95% CI)**			**78**			**68**	**50.1%**	**0.22 [-0.10, 0.55]**
Heterogeneity: Chi² = 0.00, df = 1 (P = 0.94); I² = 0%								
Test for overall effect: Z = 1.34 (P = 0.18)								
**2.1.2 Acupoint injection with herbs**								
Lin 2011	121	10.21	30	116.37	6.34	30	20.1%	0.54 [0.02, 1.05]
Xu et al 2011	109.27	16.1	15	102.68	12.75	15	10.2%	0.44 [-0.28, 1.17]
**Subtotal (95% CI)**			**45**			**45**	**30.3%**	**0.51 [0.09, 0.93]**
Heterogeneity: Chi² = 0.04, df = 1 (P = 0.83); I² = 0%								
Test for overall effect: Z = 2.36 (P = 0.02)								
**2.1.4 Moxibustion**								
Zhang 2010	113.33	5.35	30	109.1	6.84	30	19.7%	0.68 [0.16, 1.20]
**Subtotal (95% CI)**			**30**			**30**	**19.7%**	**0.68 [0.16, 1.20]**
Heterogeneity: Not applicable								
Test for overall effect: Z = 2.56 (P = 0.01)								
**Total (95% CI)**			**153**			**143**	**100.0%**	**0.40 [0.17, 0.63]**
Heterogeneity: Chi² = 2.52, df = 4 (P = 0.64); I² = 0%								
Test for overall effect: Z = 3.38 (P = 0.0007)								
Test for subgroup differences: Chi² = 2.47, df = 2 (P = 0.29), I² = 19.2%								

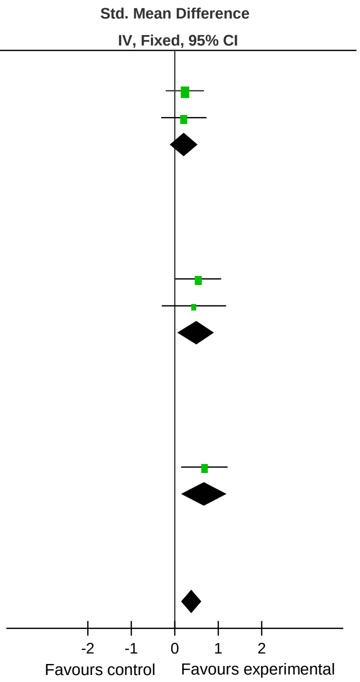

B. Platelet

Study or Subgroup	Experimental Mean	Experimental SD	Experimental Total	Control Mean	Control SD	Control Total	Weight	Std. Mean Difference IV, Fixed, 95% CI
**2.2.1 Acupoint Needle insertion**								
Ding 2011a	213.96	96.69	48	191.07	77.38	38	28.9%	0.26 [-0.17, 0.68]
Xu 2012	196.9	40.2	30	183.33	39.94	30	20.3%	0.33 [-0.18, 0.84]
**Subtotal (95% CI)**			**78**			**68**	**49.1%**	**0.29 [-0.04, 0.62]**
Heterogeneity: Chi² = 0.05, df = 1 (P = 0.82); I² = 0%								
Test for overall effect: Z = 1.72 (P = 0.08)								
**2.2.2 Acupoint injection with herbs**								
Lin 2011	115.57	8.08	30	113.87	7.52	30	20.5%	0.21 [-0.29, 0.72]
Xu et al 2011	178.9	41.8	15	174.3	36.7	15	10.3%	0.11 [-0.60, 0.83]
**Subtotal (95% CI)**			**45**			**45**	**30.7%**	**0.18 [-0.23, 0.60]**
Heterogeneity: Chi² = 0.05, df = 1 (P = 0.82); I² = 0%								
Test for overall effect: Z = 0.86 (P = 0.39)								
**2.2.4 Moxibustion**								
Zhang 2010	208.23	61.36	30	183.97	55.98	30	20.1%	0.41 [-0.10, 0.92]
**Subtotal (95% CI)**			**30**			**30**	**20.1%**	**0.41 [-0.10, 0.92]**
Heterogeneity: Not applicable								
Test for overall effect: Z = 1.56 (P = 0.12)								
**Total (95% CI)**			**153**			**143**	**100.0%**	**0.28 [0.05, 0.51]**
Heterogeneity: Chi² = 0.56, df = 4 (P = 0.97); I² = 0%								
Test for overall effect: Z = 2.38 (P = 0.02)								
Test for subgroup differences: Chi² = 0.46, df = 2 (P = 0.79), I² = 0%								

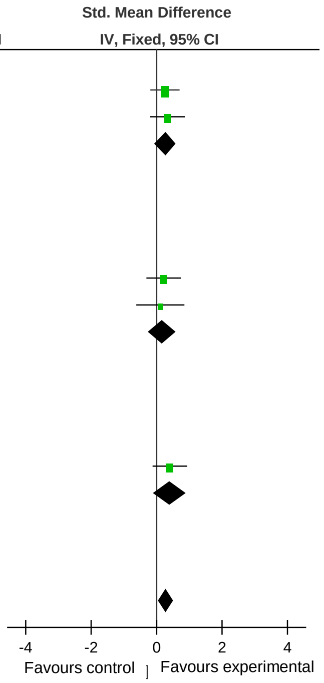

Figure S2 *(cont'd)*

C. WBC

Study or Subgroup	Experimental Mean	Experimental SD	Experimental Total	Control Mean	Control SD	Control Total	Weight	Std. Mean Difference IV, Random, 95% CI
**2.3.1 Acupoint Needle insertion**								
Ding 2011a	6.67	2.89	48	5.85	3.17	38	13.3%	0.27 [-0.16, 0.70]
Lin et al 2010	5.77	2.88	41	3.6	1.52	42	13.1%	0.94 [0.48, 1.39]
Xu 2012	6.25	0.68	30	5.46	0.61	30	12.4%	1.21 [0.65, 1.76]
**Subtotal (95% CI)**			**119**			**110**	**38.9%**	**0.79 [0.23, 1.34]**
Heterogeneity: Tau² = 0.18; Chi² = 8.11, df = 2 (P = 0.02); I² = 75%								
Test for overall effect: Z = 2.78 (P = 0.005)								
**2.3.2 Acupoint injection with herbs**								
Lin 2011	5.99	0.99	30	4.92	0.9	30	12.5%	1.12 [0.57, 1.66]
Xu et al 2011	6.34	1.63	15	5.51	1.64	15	11.1%	0.49 [-0.23, 1.22]
**Subtotal (95% CI)**			**45**			**45**	**23.6%**	**0.85 [0.25, 1.46]**
Heterogeneity: Tau² = 0.09; Chi² = 1.80, df = 1 (P = 0.18); I² = 44%								
Test for overall effect: Z = 2.78 (P = 0.005)								
**2.3.4 Moxibustion**								
Chen 2009	5.9	1.0249	30	6.06	1.169	30	12.8%	-0.14 [-0.65, 0.36]
Lin et al 2011	5.19	0.99	40	3.69	0.61	40	12.7%	1.81 [1.28, 2.33]
Zhang 2010	5.19	0.99	30	3.69	0.61	30	12.1%	1.80 [1.19, 2.41]
**Subtotal (95% CI)**			**100**			**100**	**37.5%**	**1.15 [-0.17, 2.47]**
Heterogeneity: Tau² = 1.28; Chi² = 35.21, df = 2 (P < 0.00001); I² = 94%								
Test for overall effect: Z = 1.71 (P = 0.09)								
**Total (95% CI)**			**264**			**255**	**100.0%**	**0.93 [0.44, 1.42]**
Heterogeneity: Tau² = 0.42; Chi² = 47.59, df = 7 (P < 0.00001); I² = 85%								
Test for overall effect: Z = 3.73 (P = 0.0002)								
Test for subgroup differences: Chi² = 0.25, df = 2 (P = 0.88), I² = 0%								

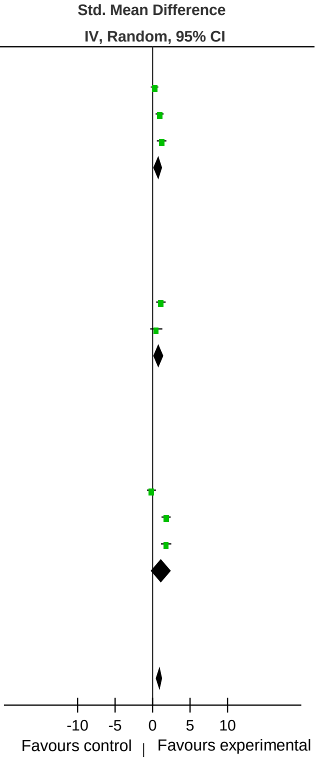

Figure S2: Bone marrow suppression in acupuncture treatment and control group. (A) Hemoglobin, (B) Platelet, and (C) ~~WBC. WBC, White blood cell.~~

Effective response of nausea and vomiting

Study or Subgroup	Experimental Events	Experimental Total	Control Events	Control Total	Weight	Risk Ratio M-H, Random, 95% CI
**11.1.1 Acupoint Needle insertion**						
Cai and Wu 2011	36	38	36	42	42.2%	1.11 [0.96, 1.28]
Ding 2011b	12	13	11	15	15.6%	1.26 [0.89, 1.77]
**Subtotal (95% CI)**		**51**		**57**	**57.8%**	**1.13 [0.99, 1.29]**
Total events	48		47			
Heterogeneity: Tau² = 0.00; Chi² = 0.50, df = 1 (P = 0.48); I² = 0%						
Test for overall effect: Z = 1.76 (P = 0.08)						
**11.1.2 Acupoint plaster application**						
Huang et al 2012	37	40	31	40	33.5%	1.19 [0.99, 1.44]
**Subtotal (95% CI)**		**40**		**40**	**33.5%**	**1.19 [0.99, 1.44]**
Total events	37		31			
Heterogeneity: Not applicable						
Test for overall effect: Z = 1.84 (P = 0.07)						
**11.1.3 Needle insertion and plaster application**						
He and Lou 2010	19	24	11	25	8.8%	1.80 [1.10, 2.93]
**Subtotal (95% CI)**		**24**		**25**	**8.8%**	**1.80 [1.10, 2.93]**
Total events	19		11			
Heterogeneity: Not applicable						
Test for overall effect: Z = 2.36 (P = 0.02)						
**Total (95% CI)**		**115**		**122**	**100.0%**	**1.21 [1.03, 1.41]**
Total events	104		89			
Heterogeneity: Tau² = 0.01; Chi² = 4.90, df = 3 (P = 0.18); I² = 39%						
Test for overall effect: Z = 2.39 (P = 0.02)						
Test for subgroup differences: Chi² = 3.34, df = 2 (P = 0.19), I² = 40.0%						

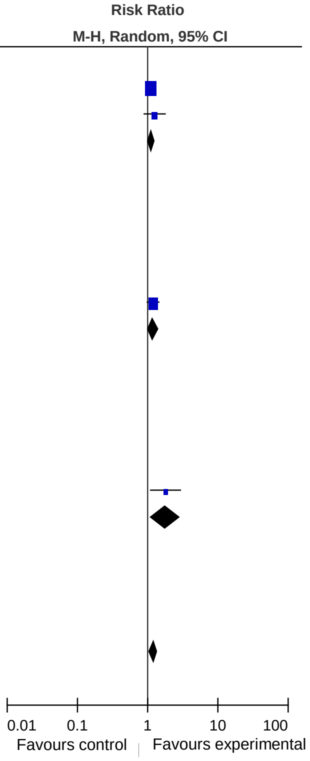

Figure S3: Effective response of nausea and vomiting in treatment and control group.
